# Carbon ion irradiation enhances the antitumor efficacy of dual immune checkpoint blockade therapy both for local and distant sites in murine osteosarcoma

**DOI:** 10.18632/oncotarget.26551

**Published:** 2019-01-18

**Authors:** Yutaka Takahashi, Tomohiro Yasui, Kazumasa Minami, Keisuke Tamari, Kazuhiko Hayashi, Keisuke Otani, Yuji Seo, Fumiaki Isohashi, Masahiko Koizumi, Kazuhiko Ogawa

**Affiliations:** ^1^ Department of Radiation Oncology, Osaka University Graduate School of Medicine, Osaka, Japan; ^2^ Department of Medical Physics and Engineering, Osaka University Graduate School of Medicine, Suita, Osaka, Japan; ^3^ Hospital of the National Institute of Radiological Sciences, National Institutes for Quantum and Radiological Science and Technology, Chiba, Japan

**Keywords:** carbon ion beam, radiation, immune checkpoint, osteosarcoma, abscopal effect

## Abstract

Carbon ion radiotherapy has been utilized even for X-ray resistant tumors. However, control of distant metastasis remains a major challenge in carbon ion irradiation. We investigated whether carbon ion irradiation combined with dual immune checkpoint blockade therapy (anti-PD-L1 and anti-CTLA-4 antibodies [P1C4]) provides anti-tumor efficacy for both local and distant sites. A mouse osteosarcoma cell line (LM8) was inoculated into both hind legs of C3H mice assigned to four groups: no treatment (NoTX), P1C4, 5.3 Gy of carbon ion irradiation to one leg (Cion), and combination (Comb) groups. In the Comb group, tumor growth delay was observed not only in the irradiated tumors but also in the unirradiated tumors. Notably, a complete response of unirradiated tumors was observed in 64% of mice in the Comb group, while only 20% of mice in the P1C4 group showed a complete response. Significant activation of immune cells was observed in the Comb group, with an increase in CD8+/GzmB+ tumor-infiltrating lymphocytes (TILs) in the irradiated tumor, and of CD8+/GzmB+ and CD4+ TILs in the unirradiated tumor, respectively. Depletion of CD8 abolished the tumor growth delay in unirradiated tumors in mice treated by Cion and P1C4. Overall survival was significantly prolonged in the Comb group. HMGB-1 release from irradiated tumors was significantly increased after Cion both *in vitro* and *in vivo*. These data suggest that carbon ion therapy enhances P1C4 efficacy against osteosarcoma in both the primary tumor and distant metastases mediated by immune activation.

## INTRODUCTION

Lymphocytes play a key role in recognizing antigens, including tumor antigens, which can lead to the elimination of cancer cells [1–3]. However, lymphocytes, as well as cancer cells, eventually send signals to suppress antitumor immunity, resulting in unlimited growth of a tumor [4–5]. Programmed Death-1 Ligand-1 (PD-L1) is an immunosuppressive protein expressed on tumor cells. The PD-1/PD-L1 pathway plays an important role in tumor immune escape [6–9]. Another signal is immunosuppressive protein Cytotoxic T lymphocyte antigen 4 (CTLA-4). Under normal conditions, the CD28/B7 pathway promotes an immune response. However, CTLA-4 binds B7 more strongly than CD28, resulting in the suppression of immune activation [10, 11]. The antitumor effect of anti-PD-L1 (P1) and anti-CTLA-4 antibodies (C4), one of the antibodies of immune checkpoint molecules, has been under investigation. Some studies have demonstrated that the combination of anti-PD-L1 and anti-CTLA-4 antibodies (P1C4) activates the immune system and induces a strong antitumor effect [12–15].

Osteosarcoma is one of the most common primary malignant tumors in children and adolescents, and is commonly treated with surgery and chemotherapy [16]. The surgery can involve amputation and may seriously affect quality of life. Although radiation therapy has been utilized for such cases because of its noninvasive features, osteosarcoma is relatively insensitive to photon beams (i.e; X-ray, gamma-ray), and radiotherapy has not been the first choice for the treatment.

Unlike to photon beams, carbon ion beams have a number of physical and biological advantages. For example, carbon ion beams provide a superior dose distribution owing to the presence of the Bragg peak, which achieves highly concentrated dose to the target with rapid dose fall-off in normal tissues [17]. Moreover, carbon ion has two to three times-stronger cell killing effect than photon beams even in hypoxic tumors and photon-resistant tumors such as osteosarcoma and melanoma because of a high probability of DNA double-strand breaks [18–21]. Recent investigations have demonstrated that particle irradiation suppresses the potential for migration and invasion of endothelial cells and malignant cells even at relatively low doses, and may prevent tumor angiogenesis and metastasis [22–25]. In fact, the number of carbon ion radiation therapy facility is growing all over the world [17].

In addition to its therapeutic effect on primary tumors, radiation therapy induces tumor regression even in unirradiated tumor sites and in distant metastases. This phenomenon is known as the “abscopal effect,” and is induced by immune modulation after irradiation [26–28]. Although the abscopal effect has been rarely seen in both preclinical models and clinical practice in radiation therapy alone [26–28], recent studies have shown that combining radiation therapy with immune checkpoint blockade is associated with a higher probability of the abscopal effect in a number of tumors [29–31]. While increasing evidence suggests that photon beam irradiation combined with immune checkpoint blockade is highly effective [29–31], the efficacy of P1C4 in combination with carbon ion irradiation for irradiated tumors and tumors out-of-radiation-field has not been reported.

Here, we investigated the anti-tumor efficacy of carbon ion irradiation combined with P1C4 for local and distant sites.

## RESULTS

### Carbon ion irradiation enhanced PD-L1 and CD80 expression on LM8 cells *in vitro*

**Figure 1:**
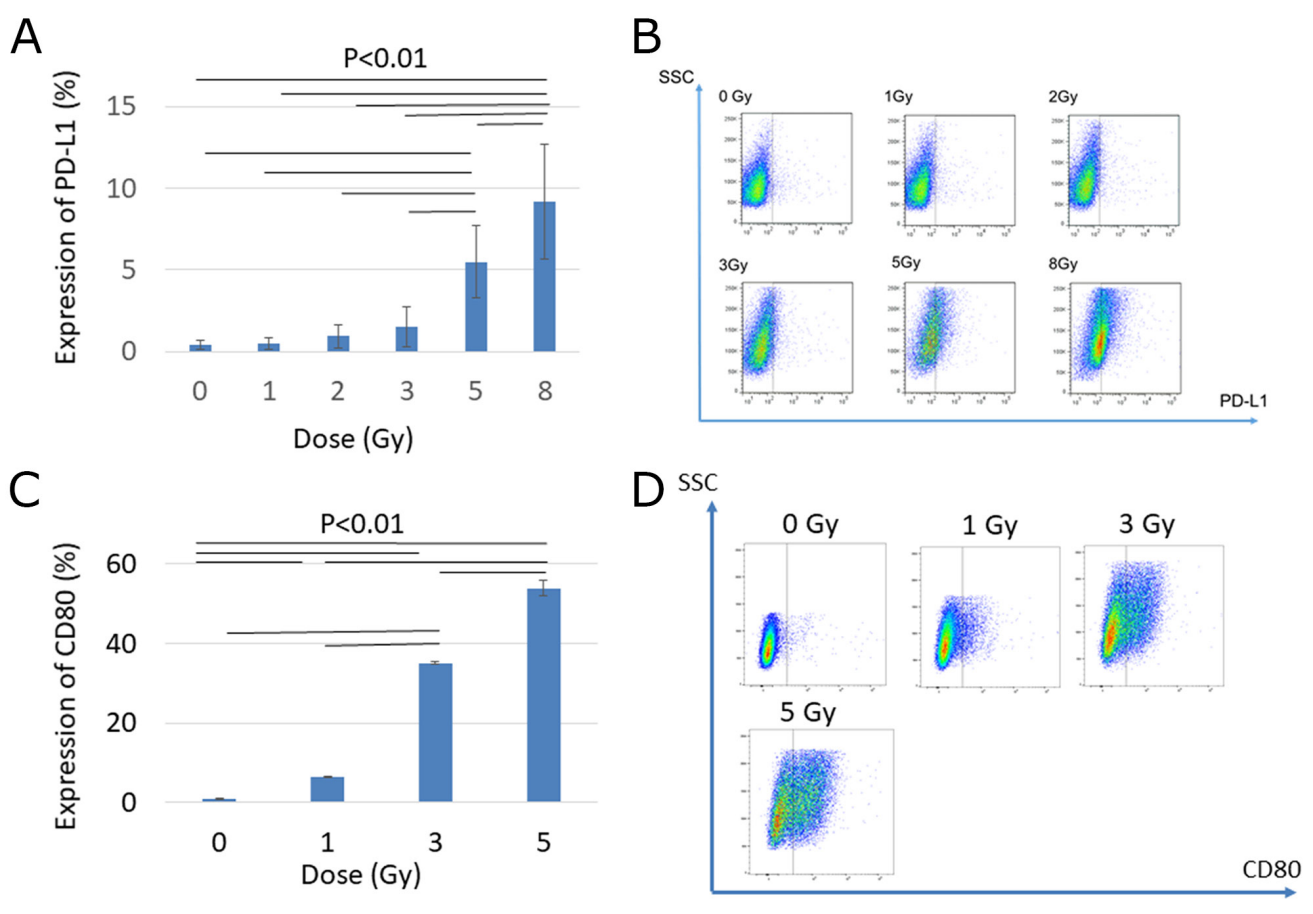
Effect of carbon ion irradiation on PD-L1 and CD80 expression changes 72 hours after irradiation **(A)** Changes in PD-L1 protein expression by flow cytometry. **(B)** Representative flow cytometric plots of PD-L1 expression. **(C)** Changes in CD80 protein expression by flow cytometry. **(D)** Representative flow cytometric plots of CD80 changes. Error bars show standard deviation. P-values were determined by Tukey's honestly significant difference tests for the comparison between each dose level. Bars represent P<0.01.

To investigate the effect of carbon ion irradiation on immune checkpoint molecules, we evaluated the protein and gene expressions of PD-L1 and CD80 after carbon ion irradiation *in vitro*. As shown in Figure 1A and 1B, PD-L1 expression was upregulated in a dose-dependent manner at 72 h after carbon ion irradiation. In particular, high-dose carbon ion irradiation (5 Gy, and 8 Gy) significantly increased PD-L1 expression compared with a low dose (1, 2, 3 Gy). Similarly, expression of CD80 increased dose-dependently, particularly at a high dose (5 Gy), as shown in Figure 1C and 1D. These results suggest that carbon ion irradiation increased immune checkpoint proteins *in vitro*, and might thereby enhance the efficacy of immune checkpoint blockade.

### Determination of equivalent dose of survival fractions in LM8 cells irradiated with a different type of ionizing radiation

**Figure 2:**
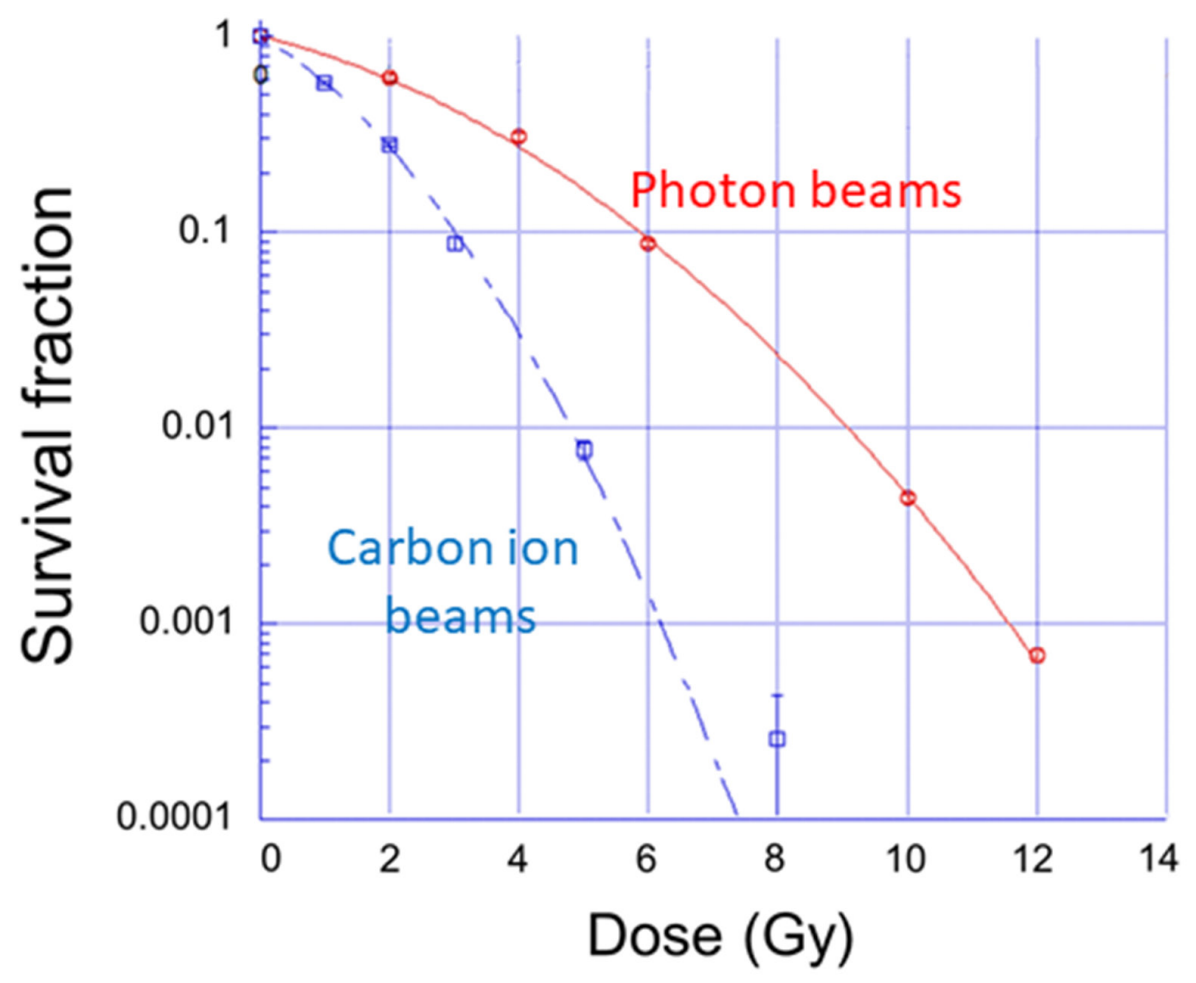
Survival fractions in LM8 cells irradiated with photon and carbon ion beams (Red line: Photon beams, blue dotted line: carbon ion beams). Results were normalized to unirradiated cells. Each bar represents the mean ± SD.

We previously demonstrated that dual immune checkpoint blockade with 10 Gy X-ray irradiation enhanced antitumor efficacy [30]. To determine the equivalent survival dose in carbon ion beams to that in photon beams in this experiment, colony formation assay was performed and a linear-quadratic (LQ) model was used to calculate the survival at a specific dose. The physical dose of 10 Gy photon beams resulted in 0.4% survival of LM8 cells. The equivalent dose required to obtain the same cell survival with carbon ion beams was found to be 5.3 Gy (Figure 2). We therefore carried out subsequent experiments with this physical carbon ion dose.

### Carbon ion irradiation combined with dual immune checkpoint blockade improves radiosensitivity of the irradiated tumor

**Figure 3:**
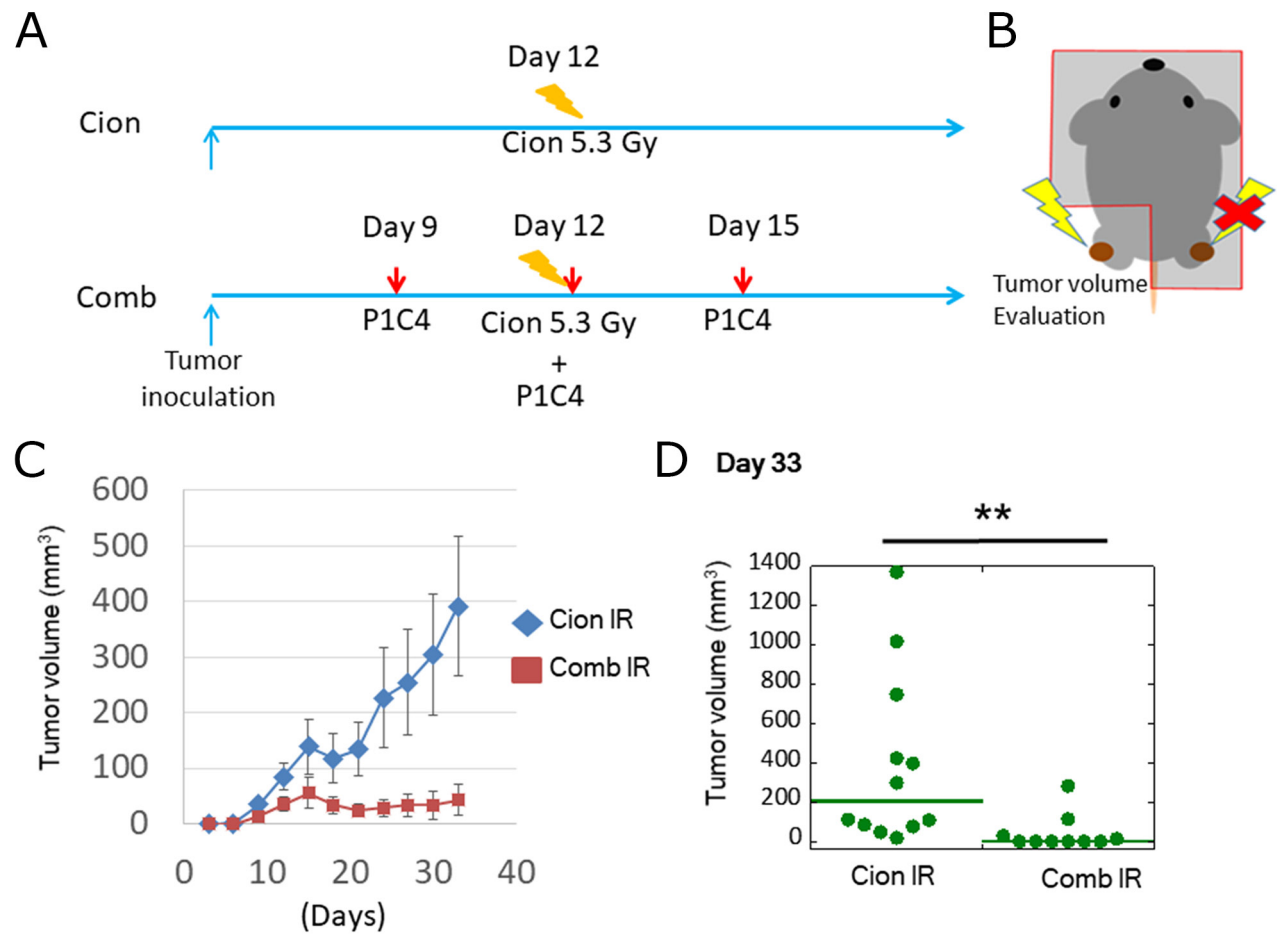
Evaluation of tumor volume change at irradiated tumor **(A)** Treatment schedule. **(B)** Scheme of mice irradiation. **(C)** Tumor growth of irradiated tumors in the Cion (N=12) and Comb groups (N=10). Each bar represents the standard error. **(D)** Quantitative analysis of tumor volume change on day 33. Green lines show median values. P-values were determined by the exact Wilcoxon two-sample test; ^**^, P<0.01.

To assess the radiosensitizing effect of dual immune checkpoint blockade, we evaluated the volume of irradiated tumors with or without P1C4. LM8 osteosarcoma cells were inoculated to both legs and only one side leg was irradiated at 5.3 Gy of carbon ion beams on day 12 (Figure 3A and 3B). Carbon ion irradiation inhibited tumor growth by day 21 but the regrowth was found after day 24 (Figure 3C). In contrast, addition of P1C4 on days 9, 12, and 15 to carbon ion irradiation enhanced the antitumor response for longer period. Of note, the combination therapy increased radiosensitivity by >2-folds (Figure 3D) on day 33. These results suggest that P1C4 worked as a radiosensitizer.

### Carbon ion irradiation combined with dual immune checkpoint blockade enhances TIL activation at the irradiated tumor

**Figure 4:**
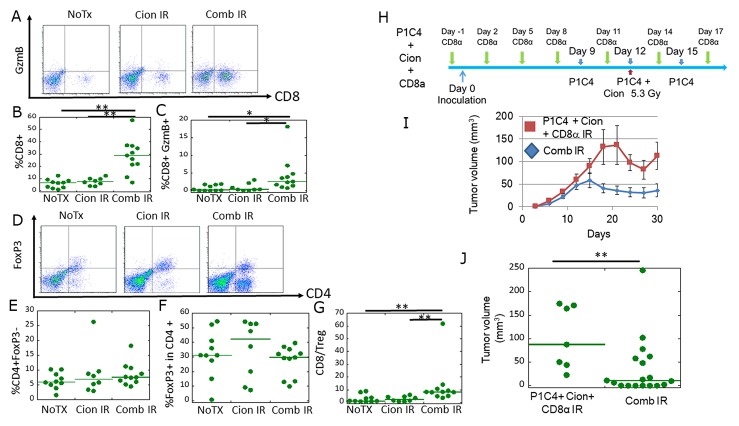
Analysis of CD8+/GzmB+ cells and CD4/Foxp3+ cells in tumor-infiltrating lymphocytes (TILs), and the antitumor immune response at a local site **(A)** Representative flow cytometry dot-plots are shown of anti-CD8 and anti-GzmB antibody-labeled T cells isolated from the tumor of NoTX (N=10), Cion IR leg (N=8), and Comb IR leg (N=11). **(B)** Quantitative data of the proportion of CD8 + TILs. **(C)** Quantitative data of the proportion of CD8 +/GzmB + TILs. **(D)** Representative flow cytometry dot-plots of anti-CD4 and anti-FoxP3 antibody-labeled TILs isolated from the tumor of NoTX, Cion IR leg, and Comb IR leg. **(E)** Quantitative data of the proportion of CD4 +, FoxP3 (helper T cell). **(F)** Quantitative data of the proportion of FoxP3+ (Treg) in CD4 TILs. **(G)** Quantitative data of the proportion of CD8+ TIL to Treg rate. Green lines represent median value. P-values were determined by Steel-Dwass test; ^**^, P<0.01. **(H)** Experimental scheme of CD8 depletion. In addition to P1C4 and Cion therapy, CD8α was injected into mice 1 day before tumor inoculation and every 3 days thereafter until day 17. **(I)** Tumor growth of irradiated tumors in the Comb group (N=18) and that those in P1C4+ Cion+ CD8α group (N=7). Each bar represents the standard error. **(J)** Quantitative analysis of tumor volume change on days 30. Green lines represent median values. P-values were determined by the exact Wilcoxon two-sample test; ^**^, P<0.01. Abbreviations: NoTX: No treatment. Cion: carbon ion irradiation. Comb: Anti-PD-L1 and anti-CTLA-4 antibodies with carbon ion irradiation. IR: Irradiation P1C4+ Cion+ CD8α: Anti-PD-L1 and anti-CTLA-4 antibodies with carbon ion irradiation, and anti-CD8 antibody.

GzmB (granzyme B) is present in NK cells and activated cytotoxic T cells and plays an important role in inducing tumor cells to undergo apoptosis [32]. Helper T cells (CD4 +, FoxP3-) are also important factors to activate immune cells including B cells, macrophage, and cytotoxic T cells [33]. In contrast, some helper T cells differentiate into regulatory T cells (Treg), in which FoxP3 is specifically expressed [34]. To investigate the differences in the immune microenvironment in irradiated tumors between the Cion and Comb groups, we assessed the expression of CD8+/GzmB+ cells and CD4+/FoxP3+ Tregs in TILs. Figure 4A shows representative flow cytometric plots of CD8+/GzmB (+) TILs in the irradiated tumors. TILs in the subset of CD8+ and GzmB+ were significantly increased in the Comb group compared with the No Tx and Cion groups (Figure 4B and 4C). In contrast, our results showed no significant differences in helper T cells (Figure 4D and 4E) and FoxP3+ cells in CD4+ cells among these treatments (Figure 4F). Accordingly, the indicator of immune activation, CD8/Treg ratio, which is defined as the ratio of the population of the CD8+ cells to that of CD4+FoxP3+ cells in the corresponding mouse, was significantly increased by the combination therapy as compared with that in the NoTx and Cion groups (Figure 4G). These results suggest that carbon ion irradiation combined with dual immune checkpoint blockade may enhance the immunity especially via CD8-mediated response at the irradiated tumor.

To test this hypothesis, we evaluated the therapeutic efficacy by administering CD8 depletion antibody *in vivo* (Figure 4H). This treatment schedule is based on a previous report by Victor et al. [29]. As shown in Figure 4I, the combination of P1C4 with carbon ion irradiation dramatically inhibited tumor growth. In contrast, CD8 depletion significantly diminished the inhibition of the tumor growth (Figure 4I and 4J). These results suggest that CD8+ TILs play an important role in the radiosensitizing effect for the irradiated tumors.

### Combination of carbon ion irradiation with dual immune checkpoint blockade enhances anti-tumor efficacy at distant site

**Figure 5:**
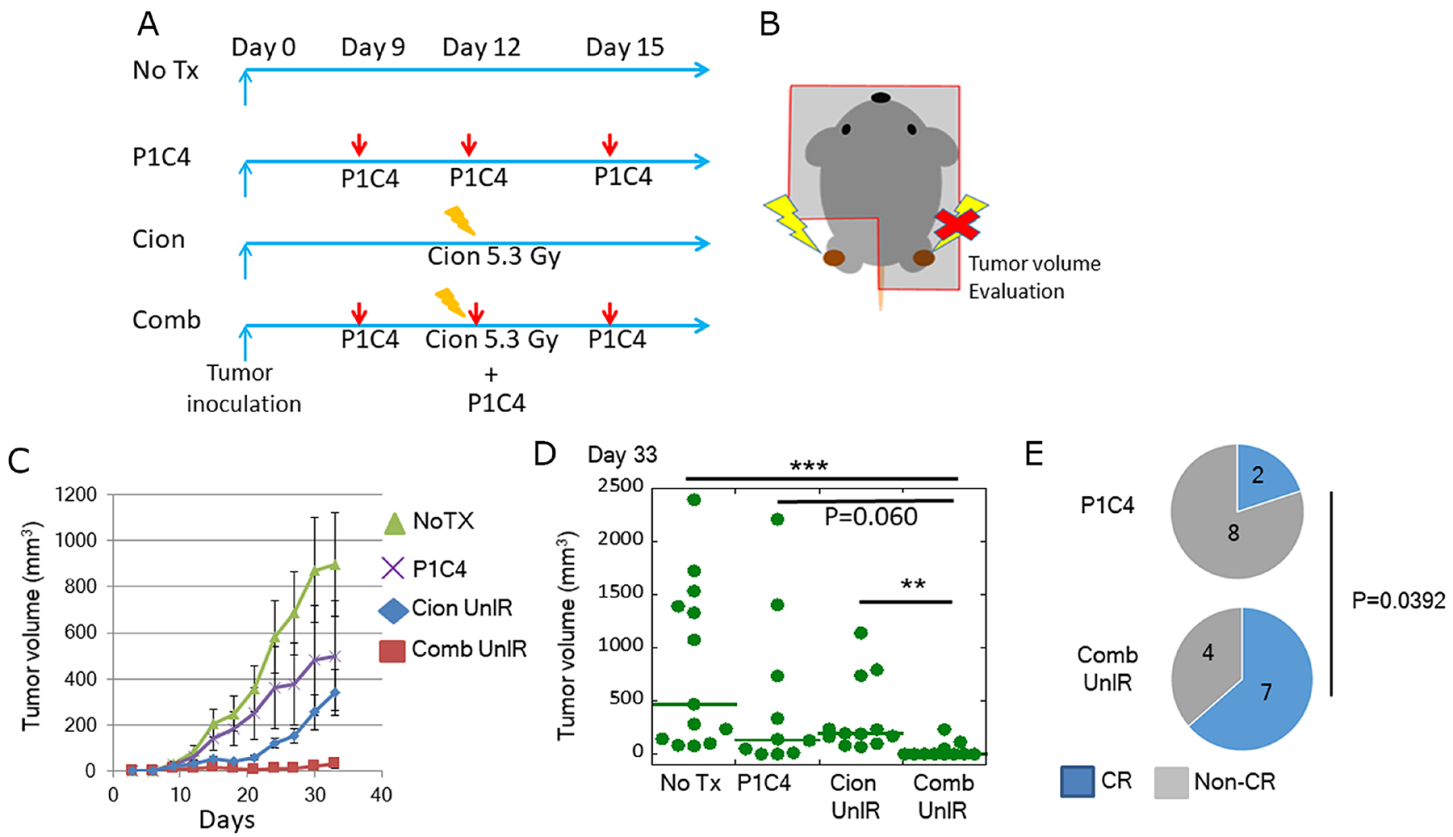
Evaluation of tumor volume change at distant tumors **(A)** Treatment schedule. **(B)** Scheme of irradiation and tumor volume evaluation. **(C)** Tumor growth in the NoTX (N=13) and P1C4 (N=10) groups, and in unirradiated tumors in the Cion (N=12) and Comb groups (N=11). Each bar represents the mean ± SE. **(D)** Quantitative analysis of tumor volume change on day 33. Green lines represent the median value. P-values were determined by Steel-Dwass test. ^**^, P<0.01, ^***^, P<0.001. **(E)** Proportion of mice with complete response. The blue part in the pie chart indicates the number of CR mice on the day at endpoint. P-values were determined by Chi-squared test. Abbreviations: NoTX: No treatment. P1C4: Anti-PD-L1 and anti-CTLA-4 antibodies. Cion: carbon ion irradiation. Comb: Anti-PD-L1 and anti-CTLA-4 antibodies with carbon ion irradiation. CR: Complete response. IR: Irradiated. UnIR: Unirradiated.

To examine whether combined therapy increases the probability of the abscopal effect, we evaluated the tumor volume change and complete response rate in unirradiated tumors (Out-of-radiation-field tumor) in mice in the NoTX, P1C4, Cion, and Comb groups (Figure 5A and 5B).

Although volume changes of the unirradiated tumor in the Cion group showed slight suppression, the addition of P1C4 to carbon ion irradiation significantly suppressed the tumor growth in comparison with that in the NoTx and Cion groups (Figure 5C). Quantitative analysis revealed that this trend continued even on day 33 (Figure 5D). Substantial decrease in the unirradiated tumor volume was observed in the Comb group as compared with that in the P1C4 group. Moreover, analysis using generalized linearity model showed that the addition of carbon ion irradiation to P1C4 could synergistically enhance the efficacy of the unirradiated tumors (P < 0.001). Although unirradiated tumor in Mice in the NoTX and Cion groups did not experienced CR, the CR rate in the Comb group was significantly increased (P=0.0392), as shown in Figure 5E. Specifically, only 2 of 10 mice (20%) in the P1C4 group experienced CR, versus 7 of 11 mice (64%) in the Comb group, suggesting that combination of carbon ion irradiation with dual immune checkpoint blockade enhanced the abscopal effect and provided anti-tumor efficacy at a distant site.

### Combination therapy enhanced CD8+ TIL activity and increased CD4+ TILs in unirradiated tumors

We next investigated whether tumor growth delay in the unirradiated tumors was mediated by immune activation by analyzing the expression of CD8+/GzmB+ cells and CD4/Foxp3+ cells in TILs by flow cytometry.

**Figure 6:**
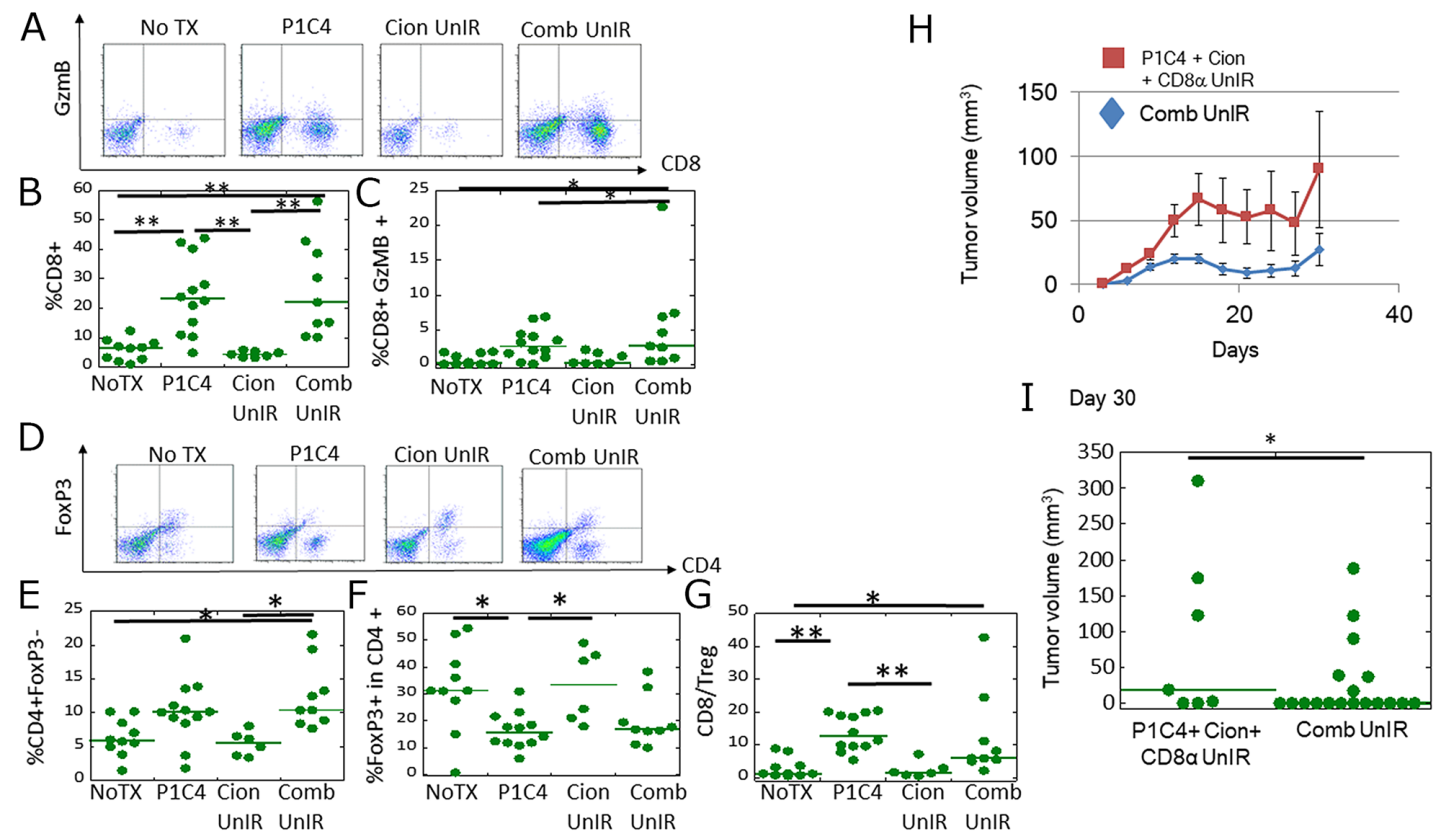
Analysis of CD8+/GzmB+ cells and CD4/Foxp3+ cells in tumor infiltrating lymphocytes (TILs) at a distant site **(A)** Representative flow cytometry dot-plots showing anti-CD8 and anti-GzmB antibody-labeled TILs isolated from the unirradiated tumors in the NoTX (N=10), P1C4 (N=12), Cion (N=7) and Comb (N=9) groups. **(B)** Quantitative data of the proportion of CD8+ and GzmB+ TILs. **(C)** Quantitative data of the proportion of CD8+/GzmB+ TILs. **(D)** Representative flow cytometry dot-plots showing anti-CD4 and anti-Foxp3 antibody-labeled T cells isolated from the unirradiated tumors in the NoTX (N=10), P1C4 (N=12), Cion (N=6) and Comb groups (N=9). **(E)** Quantitative data of the proportion of CD4 +, Foxp3- (helper T cell). **(F)** Quantitative data of the proportion of Foxp3+ (Treg) in CD4 TILs. **(G)** Quantitative data of the proportion of CD8 TILs to Treg ratio. Green lines represent median values. P-values were determined by Steel-Dwass test; ^*^, P<0.05, ^**^, P<0.01. Note that the flow cytometry data of the NoTx group were shared with those in Figs. 4 because the experiment was conducted on the same day under exactly the same procedures and the same conditions. **(H)** Tumor growth of irradiated tumors in the Comb group (N=18) and that those in P1C4+ Cion+ CD8α group (N=7). Treatment was conducted as shown in Figure 4H. Each bar represents the standard error. **(I)** Quantitative analysis of tumor volume change on days 30. Green lines represent median values. P-values were determined by the exact Wilcoxon two-sample test. ^*^, P<0.05. ^**^, P<0.01. Abbreviations: NoTX: No treatment; P1C4: Anti-PD-L1 and anti-CTLA-4 antibodies; Cion: carbon ion irradiation; and Comb: Anti-PD-L1 and anti-CTLA-4 antibodies with carbon ion irradiation. P1C4+ Cion+ CD8α: Anti-PD-L1 and anti-CTLA-4 antibodies with carbon ion irradiation, and anti-CD8 antibody.

As shown in Figure 6A-6C, a significant increase in CD8+ and CD8+/GzmB+ TILs was observed in the P1C4 and Comb groups compared with the NoTX group. Assessment of Treg in CD4+ TILs showed that the percentage of Tregs was significantly decreased in the P1C4 group compared with the NoTX and Cion groups (Figure 6D, 6F). Importantly, a significant increase in CD4+ FoxP3- TILs was observed only in the Comb group compared with NoTX and Cion groups (Figure 6D, 6E). Accordingly, CD8/Treg ratio was increased in both P1C4-treated groups (Figure 6G). These results suggest that the abscopal effect may be related with the activation of CD8 TILs and increase in CD4+ TILs.

To investigate whether the antitumor effect in unirradiated tumors was mediated by CD8+ T cells, we evaluated the therapeutic efficacy by administering CD8 depletion antibody as shown in Figure 4H. Unirradiated tumor in the P1C4+ Cion + CD8α group grew rapidly compared with the Comb group (Figure 6H). Because of the CD8α administration before day 9 in the P1C4+ Cion + CD8α group, the unirradiated tumor volume in the P1C4 + Cion + CD8α group was 1.7-fold greater than that in the Comb group at the initiation of the treatment on day 9. As shown in Figure 6I, however, this difference was significantly augmented to 3.3-fold on day 30. These results suggest that CD8+ TILs play an important role in the antitumor effect on the distant tumor.

### Carbon ion irradiation combined with dual immune checkpoint blockade inhibits distant metastasis and provide a survival benefit

**Figure 7:**
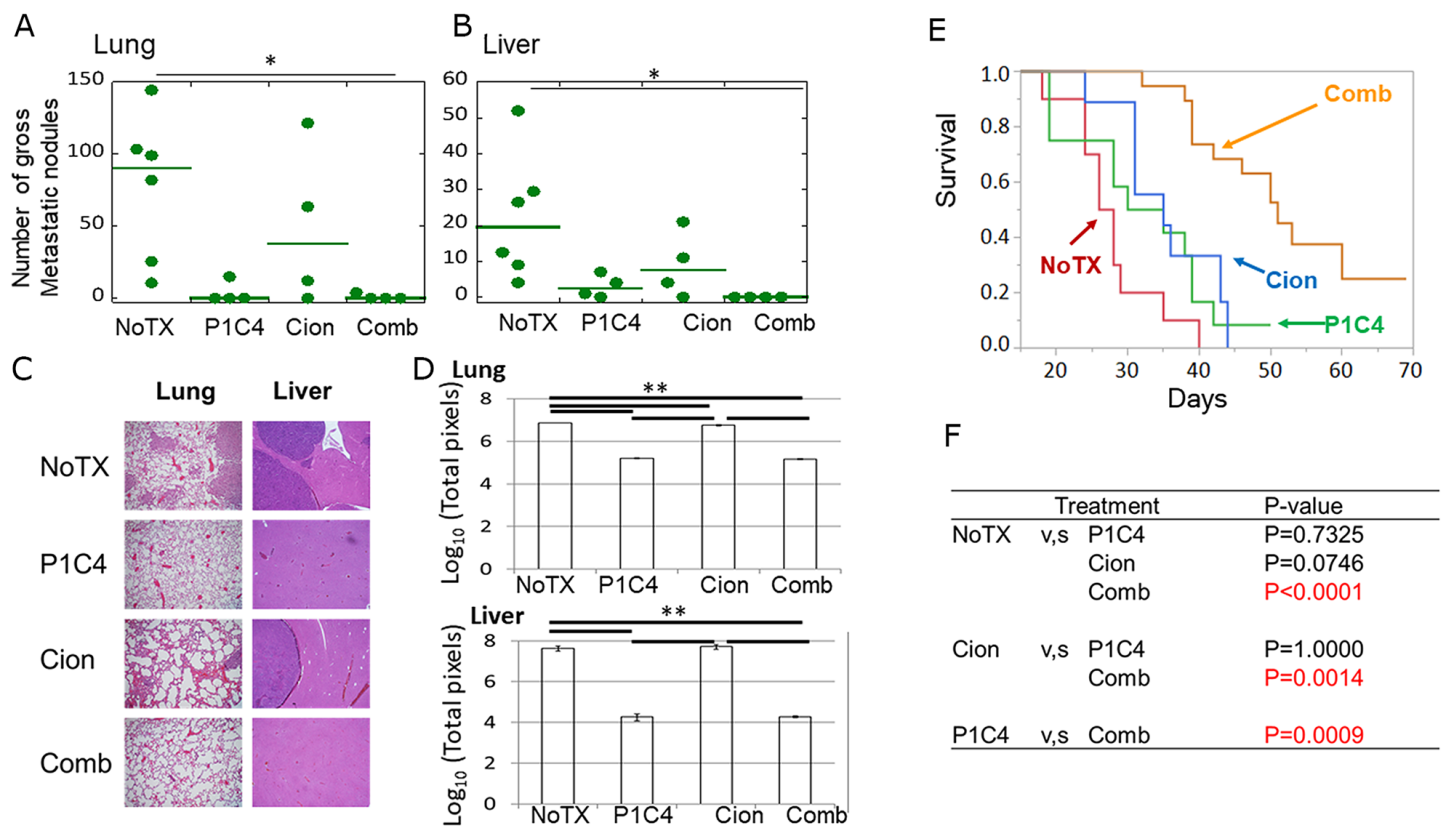
Evaluation of tumor metastasis and overall survival **(A)** Quantitative analysis of the number of gross metastatic nodules in the lung in the NoTx (N=6), P1C4 (N=4), Cion (N=4), and Comb groups (N=4). Green lines mean median values. P-values were determined by Turkey's honestly significant difference test. **(B)** Quantitative analysis of number of gross metastatic nodules in the liver in the NoTx (N=6), P1C4 (N=4), Cion (N=4), and Comb groups (N=4). Green lines represent median values. P-values were determined by Turkey's honestly significant difference test; ^*^, P<0.05. **(C)** Hematoxylin-eosin staining for the lung and liver (Magnification, lung; x100, liver; x40). **(D)** Quantitative evaluation of the micrometastasis was performed by counting the total number of pixels in tumors identified by a microscope under 100x magnification through the entire regions of a section by moving the microscope stage. Three slides were analyzed per group. **(E)** Overall survival of mice in the NoTX (N=10), P1C4 (N=12), Cion (N=9), and COMB (N=19) groups. **(F)** Comparison of overall survival between NoTX, P1C4, Cion, and Comb groups. P-values were determined by the Log-rank test with adjustment by Bonferroni correction. Abbreviations: NoTX: No treatment; P1C4: Anti-PD-L1 and anti-CTLA-4 antibodies; Cion: carbon ion irradiation; and Comb: Anti-PD-L1 and anti-CTLA-4 antibodies with carbon ion irradiation. CR: complete response.

To assess whether carbon ion irradiation combined with P1C4 is effective for the suppression of distant metastasis, we evaluated the number of metastases in the lungs and liver of mice on day 31 after tumor inoculation. As seen in Figure 7A, and 7B, the number of gross metastatic nodules in the liver and lung was significantly decreased in the Comb group compared with the NoTX group. The analysis of micrometastases confirmed the trend of gross metastatic nodules, wherein the mice in the P1C4 and Comb groups showed significantly suppressed metastasis compared with that in the mice in the NoTx and Cion groups (Figure 7C, 7D). Importantly, combination therapy significantly improved overall survival compared with the P1C4 treatment group, although overall survival in the P1C4 group did not significantly differ from that in the NoTX group (Figure 7E and 7F). Median survival in the P1C4, Cion, and Comb groups was 32.5, 35, and 51 days, respectively, suggesting that combination therapy is the most effective treatment option for osteosarcoma.

### Carbon ion irradiation increases the release of HMGB-1 *in vitro* and *in vivo*

**Figure 8:**
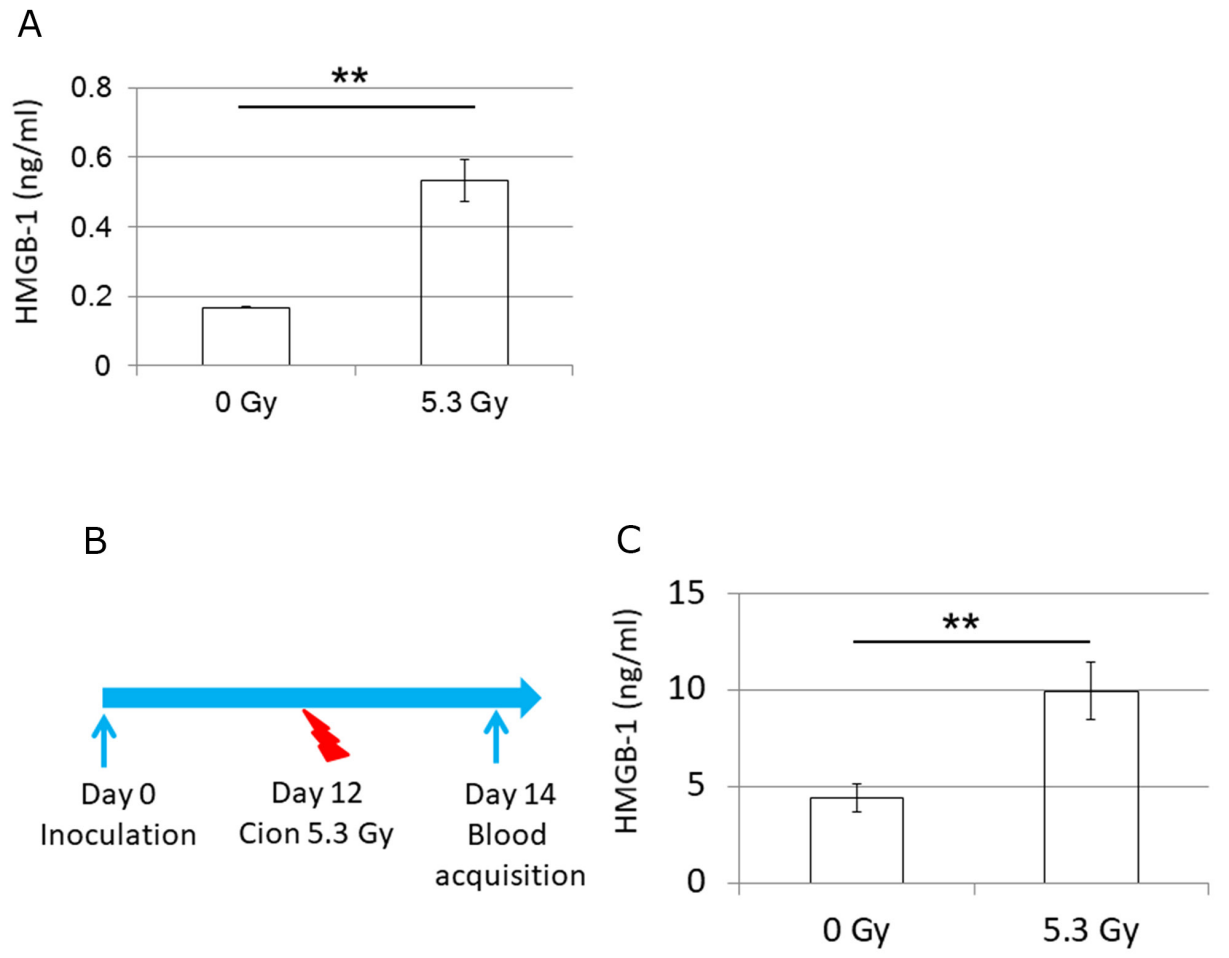
Release of HMGB-1 *in vitro* and *in vivo* **(A)** Release of HMGB-1 to cell culture supernatants from treated (N=3) or untreated LM8 cells (N=3). **(B)** Treatment schedule for the HMGB-1-release experiment *in vivo*. **(C)** Release of HMGB-1 to serum from treated (N=7) or untreated mice (N=4). P-values were determined by Student's *t*-test; ^**^, P<0.01.

Because carbon ion irradiation may contribute to the abscopal effect mediated by CD8 T cells, and the prolonged overall survival, we next investigated whether carbon ion irradiation induces the immunogenic cell death, thereby releasing HMGB-1 that maturate dendritic cells and activate helper T cells and killer T cells [35, 36]. First, we plated LM8 cells to T25 flasks 12 hours before irradiation and then unirradiated (N=3) or irradiated by 5.3 Gy (N=3) of carbon ion beams. Analysis of HMGB-1in the cell culture supernatants collected 48 hours after carbon ion irradiation revealed >3-fold increase of HMGB-1 from the irradiated cells compared with untreated cells (Figure 8A). To investigate whether the release of HMGB-1 from tumors is reflected on LM8 bearing mice, we irradiated tumors on one side leg and collected blood 2 days after carbon ion irradiation (Figure 8B). HMGB-1 release into serum in the irradiated mice (N=7) was increased by > 2 folds compared to untreated mice (N=4) (Figure 8C), suggesting that the carbon ion irradiation effect on immunogenic cell death was propagated to peripheral blood.

## DISCUSSION

Standard therapy for osteosarcoma is the combination of chemotherapy and surgery [37]. Due to the radio-resistance of osteosarcoma, photon beam radiation therapy is used in only limited cases [38]. We have evaluated the efficacy of photon therapy combined with dual immune checkpoint blockade for osteosarcoma and were the first to show that combination therapy induces a higher rate of abscopal effect than photon therapy only, and suppressed the growth of unirradiated tumors and distant metastases [30]. Carbon ion beams are effective even against X-ray-resistant tumors and demonstrate substantial efficacy against localized osteosarcoma [17, 18]. In contrast, the efficacy of carbon ion irradiation in combination with dual immune checkpoint therapy has been totally unclear, and the abscopal effect with carbon ion irradiation has been reported in a single clinical case report only [39]. To our knowledge, our present study is the first report to demonstrate that carbon ion irradiation combined with dual immune checkpoint blockade therapy enhances anti-tumor efficacy and induces the abscopal effect using preclinical model.

First, we found that addition of P1C4 to carbon ion irradiation provided radiosensitizing effect (Figure 3). We also found that depletion of CD8 significantly reduced this radiosensitizing effect, suggesting that the radiosensitizing effect of carbon ion irradiation was mediated by CD8+T cells. In addition, several researchers reported that expression of PD-L1 increased with photon beam irradiation in a dose-dependent manner, and that this effect is strongly related to therapeutic effect of PD-L1 and PD-1 antibody [40–43]. Sato et al. reported that PD-L1 expression was upregulated in various cancer cells in response to DNA double-strand break after X-ray irradiation [43]. Similarly, as shown in Figure 1A, expression of PD-L1 was increased by carbon ion irradiation. Thus, carbon ion irradiation combined with P1C4 may have efficiently suppressed irradiated tumor growth by blocking the PD-L1/PD-1 pathway.

Second, we found that the abscopal effect by carbon ion irradiation was significantly enhanced by the addition of P1C4. In previous studies, similar immune activation was observed in melanoma and osteosarcoma xenografts using photon beams combined with P1C4 [29–30]. Our flow cytometric analysis revealed that CD8+/GzmB+ TILs, and CD4+ TILs were significantly increased in the Comb compared with the NoTX group. Thus, activation of CD8+/GzmB+ and CD4+ TILs may contribute to the therapeutic effect. Indeed, CD8 depletion abolished the antitumor efficacy in unirradiated tumors even in combination therapy (Figure 6H and 6I).

In the present study, the expression of CD4+FoxP3- TILs was significantly increased only in the unirradiated tumors, and not in the irradiated tumors, after the combined therapy. Because lymphocytes are highly radiosensitive [44], transient decrease in T cells occurs in the irradiated tumors. Although there is no report yet on the dynamic alteration of the microenvironment by carbon ion irradiation, Dovedi et al. reported that X-ray irradiation to tumors lead to a transient reduction in the expression of both the CD8+ and CD4+ cells, and re-accumulation of CD8+ cells was observed after 7 days of irradiation, which was faster than that of CD4+ cells [45]. In our study, we observed no increase in the CD4+ FoxP3-cells in the irradiated tumors after 5 days of irradiation. Taken together, it seems that re-accumulation of CD4+FoxP3- cells occurs at the later time point (i.e., after 10 days).

Chemokines also play an important role in the migration and requirement of CD8 and CD4+ T cells to tumors [46]. For example, increased infiltration of CD8+ and CD4+ T cells was associated with CXCL16 and its receptor CXCR6 expression in colorectal cancers [47]. Although no reports have been published on the effect of carbon ion irradiation on chemokine secretion, a previous report demonstrated that X-ray irradiation to a tumor strongly induced CXCL16 [48], which may lead to increased recruitment of T cells at the distant site. Therefore, we presume that the secretion of chemokines may contribute to the induction of CD4+ FoxP3- T cell in the unirradiated tumors. Further studies are necessary to elucidate the effect of carbon ion irradiation on chemokine secretion and tumor microenvironment dynamics associated with T cell recruitment.

X-ray irradiation is known to elicit immunogenic cell death which acts as an activator of dendritic cells [35]. This cell death is associated with the activation of danger signaling pathways eliciting emission of damage associated molecular patterns (DAMPs) such as expression of heat shock proteins (HSP70 and HSP90), and endoplasmic reticulum (ER) chaperone calreticulin on the cell membrane, and release of ATP and HMGB from dying tumor cells [35, 49–51]. For example, HMGB-1 plays an important role in antigen-specific T cell response [51]. Specifically, the released HMGB1 binds to toll-like receptor 3 on dendritic cells, leading to antigen presentation and CTL activation. Although previous research demonstrated that stimuli including X-ray irradiation, chemotherapy, and hyperthermia induced immunogenic cell death [35], the effect of carbon ion irradiation has not been fully elucidated. Yoshimoto et al. and Onishi et al. reported that the release of HMGB-1 from human cervical cancer and esophagus cancer cell lines was increased not only by X-ray beams but also by carbon ion beams *in vitro* [52, 53]. Similarly, our data also revealed that carbon ion irradiation significantly promoted the release of HMGB-1 from irradiated osteosarcoma *in vitro* and *in vivo*. Furthermore, increased release of HMGB-1 was confirmed *in vivo* (Figure 8C). Taken together, our results suggest that carbon ion- induced DAMP may be associated with the CD8-mediated radiosensitizing effect and the abscopal effect.

Finally, we examined whether carbon ion irradiation with P1C4 contributes to distant metastasis and overall survival. The evaluation of the lung and liver metastasis on day 20 revealed no gross metastatic nodules in both the P1C4 and the Comb groups (data not shown). Because the dead mice in the survival experiments had lung and liver metastases, we selected day 31, which is approximately equal to the median survival of the P1C4 group, to examine the time course of the distant metastasis development. We found that while gross metastasis was slightly decreased in the P1C4-treated group in the lung and liver, it was significantly decreased in the Comb group compared with the NoTX group (Figure 7A, and 7B). Although treatment of P1C4 alone shows a trend to decrease distant metastasis, overall survival was significantly prolonged only in the Comb group, indicating that P1C4 therapy was effective only in early time point and was unable to sufficiently suppress tumor growth at the primary site. These results suggest that addition of carbon ion irradiation to P1C4 is promising therapeutic option for osteosarcoma.

In conclusion, our study demonstrated that carbon ion irradiation combined with anti-PD-L1 and anti-CTLA4 therapy enhanced antitumor immunity and the inhibitory effect against tumor growth in the primary tumor and distant metastases in osteosarcoma. Combination therapy also dramatically increased survival days. This report provides the first evidence that the abscopal effect occurs with carbon ion, as it also does with photon beam, and is enhanced by administration of P1C4. Combination therapy may be an effective new treatment strategy for osteosarcoma. Further studies are required to explore the mechanism of induction of the abscopal effect, as well as optimal radiation and drug administration regimens to induce the abscopal effect for other cell lines.

## MATERIALS AND METHODS

### Cell culture and reagents

A mouse osteosarcoma cell line, LM8 was purchased from RIKEN (Tokyo, Japan) which was confirmed free of contamination by Mycoplasma by Central Institute for Experimental Animals Monitoring Center in Kanagawa, Japan. LM8 cells were maintained in Dulbecco modified Eagle medium (DMEM) with 10% fetal bovine serum, penicillin, streptomycin, and L-glutamine (Thermo Fisher Scientific, Waltham MA, USA) at 37°C in a humidified atmosphere of 5% CO_2_.The immune checkpoint blockade anti-PD-L1 antibody (Clone: 10F.9G2), anti-CTLA-4 antibody (Clone: 9H10), and anti-mouse CD8 antibody (Clone: 2.43) were purchased from Bio X Cell (West Lebanon NH, USA).

### Mouse experiment

Seven-to-eight-week-old C3H/HeNJcl mice were purchased from Nihon-Clea (Tokyo, Japan) and maintained in an area confirmed to be pathogen-free by a microbiological examination conducted at least twice a year in HIMAC (Heavy Ion Medical Accelerator in Chiba). All experimental procedures were approved by National Institutes for Quantum and Radiological Science and Technology (Approved number; 16-2001-4).

The treatment scheme was performed as previously described in our photon beam experiment [30]. Briefly, mice were injected with 3.0 × 10^5^ LM8 cells to both legs, and then assigned to one of four groups: no treatment (NoTX), anti-PD-L1 and anti-CTLA-4 antibodies alone (P1C4), carbon ion irradiation alone (Cion), and a combination (Comb) group receiving both antibodies and carbon ion irradiation. We analyzed mice having a tumor volume of up to 100 mm^3^ at initiation of the 1^st^ treatment. Mice in the P1C4 and Comb groups were treated with intraperitoneal injection of 150 ug anti PD-L1 antibody and 150 ug of the anti CTLA-4 antibody on days 9, 12, and 15. Mice in the Cion and Comb groups received carbon ion irradiation to one leg on day 12. Specifically, mice were immobilized with 30 mg/kg of pentobarbital by intraperitoneal injection and fixed on a Lucite panel. The carbon ion beam was formed to be an irradiation field of 3 cm width × 10 cm length with brass shields. A tumor in one leg was set inside the field so that the surface of the Lucite panel was at the center of 6 cm SOBP. The tumor on one leg was irradiated at 5.3 Gy in a single fraction with 290 MeV carbon ion beams in HIMAC.

For the CD8 T cell-depletion experiment, mice were treated with P1C4 and carbon ion beams with or without 120 μg of anti-CD8 antibody (CD8α) which was intraperitoneally injected 1 day before tumor inoculation and every 3 days thereafter until day 17, in accordance with Victor et al. [29].

Tumor volume was calculated with the following formula: L × W^2^ × 0.52

where L was the longest diameter and W was orthogonal to L. Average volume for 3 days were binned.

The lung and liver from the mice in the No Tx (N=6), P1C4 (N=4), Cion (N=4), and Comb groups (N=4) were harvested for metastasis analysis on day 31. Surface metastatic nodules in all pulmonary and liver lobes were counted using a stereoscopic microscope (SZX12; Olympus, Tokyo, Japan) with the magnification of 7x, as reported previously [22]. Paraffin sections from these samples were stained with hematoxylin and eosin. To evaluate micrometastasis, the tumors that were identifiable by a microscope (Intelligent Microscope, Olympus, Tokyo, Japan) at 100x magnification were contoured through the entire regions of a section by moving the microscope stage and the number of pixels of all contoured tumors was summed. Three slides were analyzed per group.

A mouse survival study was performed as described previously [30]. Mice from the tumor volume study and metastasis assay cohorts were assigned to the NoTX, P1C4, Cion, and Comb groups. An event was defined as an endpoint when the mice encountered any of the humane endpoint criteria [9], or when the tumor reached ≥ 15 mm in longest dimension. Survival was calculated using the Kaplan-Meier method, and p-values were calculated with the log-rank test and adjusted by Bonferroni correction.

### *In vitro* photon beam irradiation

To determine the relative biological effectiveness (RBE) of carbon ion beams to photon beams, T25 culture flasks of LM8 cells were irradiated with photon beams using a Gamma cell 40 Exactor (^137^Cs, γ-ray, 0.66 MeV, 0.85 Gy/min) at the Department of Radiation Oncology, Osaka University Graduate School of Medicine.

### *In vitro* carbon ion irradiation

For *in vitro* carbon ion beam irradiation, T25 culture flasks were irradiated with 290 MeV horizontal carbon ion beams at the National Institute of Radiological Science, Chiba, Japan. The culture surface was placed at the center of a 6 cm spread-out Bragg peak where the Linear Energy Transfer is approximately 50 KeV/μm.

### Colony formation assay

LM8 cells were irradiated by γ-ray doses of 2, 4, 6, 10, and 12 Gy, and by carbon ion beams doses of 1, 2, 3, 5, and 8 Gy. These X-ray and carbon ion doses were determined so that the number of survived colony was 60-100. Immediately after irradiation, cells were washed in phosphate-buffered saline (PBS, Thermo Fisher Scientific, Massachusetts, USA), harvested with 0.05% trypsin-EDTA (Thermo Fisher Scientific, Massachusetts, USA), and seeded onto culture dishes. Fourteen days after culturing, these cells were fixed with 10% formalin and stained with 0.04% crystal violet solution. After staining, we counted each colony and calculated surviving fractions (SF). These results were analyzed using the linear-quadratic (LQ) model that quantitatively describes the response to ionizing irradiation in terms of clonogenic survival, which express SF = exp (-α × D - β × D^2^). D denotes radiation dose.

### Flow cytometry

Cells for *in vitro* PD-L1 and CD80 expression analysis were irradiated by carbon ion beams. At 3 days after irradiation, cells were reacted with anti-PD-L1-PE antibody (Clone: MIH5, BD Pharmagen, NJ, USA) and anti-CD80-FITC antibody (Clone: 16-10A1, BD Pharmagen, NJ, USA).

To analyze the proportion of CD8+ and GzmB+ T cells and Treg, mice were euthanized on day 20 after tumor inoculation. Irradiated and unirradiated tumors were harvested. The removed tumors were minced in HBSS supplemented with 1% BSA. Dissociation was done in HBSS supplemented with 0.5 mg/mL collagenase IV (Sigma Aldrich, Tokyo, Japan), 200 μg/mL DNase (Sigma Aldrich) and 1% BSA on a shaker at 37°C on a shaker for 30 min as described previously [30, 54]. For Fc block, anti-mouse CD16/32 antibody (Biolegend, CA, USA) was added for 10 min at room temperature prior to reaction with CD8 or CD4 antibodies. CD8 and GzmB analysis in TILs was done using anti-mouse rat CD8-APC antibody (Clone: 53-6.7, eBioscience, San Diego, USA) and anti-mouse Granzyme-B-PE (Clone: NGZB, eBioscience), respectively. Treg analysis in TILs was done using anti-mouse rat CD4-APC (Clone: RM4-5, eBioscience), anti-mouse rat FoxP3-PE (Clone: FJK-16s, eBioscience) antibodies, as well as FoxP3/Transcription Factor Staining Buffer Set (eBioscience). Each antibody was diluted 1:80. All procedures during sample preparation were performed according to the respective manufacturer's instructions.

Stained cells were analyzed with a FACS Verse™ (BD, NJ, USA). Gating to identify lymphocytes was performed using splenocytes, and the percentage of CD8 TILs or Tregs (CD4+ and FoxP3+) [28, 33] was calculated. Analysis was conducted using Flow Jo ver.8 (Tommy Digital Biology, Tokyo, Japan)

### HMGB-1 detection

LM8 cells in T25 flasks were irradiated by carbon ion beams. The cell culture medium was change to the 2.5 ml of the fresh medium immediately after irradiation. The cell culture supernatants were collected 48 hours after irradiarion. Enzyme-linked immunosorbent assay was performed using the Mouse/Rat HMGB1 ELISA kit (Arigo biolaboratories, Taiwan) to detect an amount of release of HMGB-1.

### Statistics

Tumor volume difference in irradiated tumors between Cion and Comb groups was analyzed by the exact Wilcoxon two-sample test. For the multiple comparison of tumor volume change in the unirradiated tumors, and TILs among the NoTX, P1C4, Cion, and Comb groups, the Steel–Dwass test was used. To evaluate whether the therapeutic efficacy for unirradiated tumors was additive or synergistic in the Comb groups, generalized linearity model was applied. PD-L1 and CD80 expression *in vitro* between each dose level, and number of metastases between each treatment group were compared using Tukey's honestly significant differences test. Tumor complete response (CR) rate was compared using Chi-squared test. Overall survival between the NoTX, P1C4, Cion, and Comb groups was compared using the log rank test. P-values were adjusted with Bonferroni correction. Differences in the tumor volume in the CD8 depletion experiments were analyzed by the exact Wilcoxon two-sample test. Differences in the HMGB-1 experiments were analyzed using two-tail Student's *t*-test.

## Abbreviations

TIL: tumor infiltrating lymphocyte
GzmB: granzyme B
Cion: carbon ion irradiation
Comb: anti-PD-L1 and anti-CTLA-4 antibodies with carbon ion irradiation
NoTX: no treatment
P1C4: anti-PD-L1 and anti-CTLA-4 antibodies
CR: complete response. P1C4+ Cion+
CD8α: anti-PD-L1 and anti-CTLA-4 antibodies with carbon ion irradiation and anti-CD8 antibody
IR: irradiated
UnIR: unirradiated.
